# Supplementary material for: Letermovir Primary Prophylaxis in High-Risk Hematopoietic Cell Transplant Recipients: A Matched Cohort Study
Source: Vaccines (Basel). 2021 Apr 12;9(4):372. doi: 10.3390/vaccines9040372 (PMC8069238; doi:10.3390/vaccines9040372)
Supplement: Supplementary file 1 [file vaccines-09-00372-s001.pdf]

Table S1. Primary and secondary clinical outcomes between cases and controls during the first 180 days post study inclusion by study group[1]

Variables	CMV D-R+			CMV R+, GvHD+		
	**Cases** *N*: **16 (%)**	**Controls N: 32 (%)**	**P-value**	**Cases N: 10 (%)**	**Controls N: 20 (%)**	***P*-value**
**Primary outcome**[1]						
csCMV infection[2]	5 (31.1)	26 (81.3)	0.001	4 (40.0)	17 (85.0)	0.01
>1 csCMV infection[3]	2 (12.5)	21 (65.6)	0.001	1 (10.0)	10 (50.0)	0.05
**Secondary outcomes**[1]						
**CMV treatment duration**[4]	47.8 (15, 104)	85.2 (8, 155)	0.02	30 (22, 40)	69 (21, 142)	0.05
Ganciclovir	24 (14, 34)	23.6 (5, 39)	0.98		24.8 (8, 67)	NA
Valganciclovir	27.8 (8, 70)	62.9 (14, 112)	0.02	26.8 (17, 40)	49 (14, 142)	0.19
Foscarnet	26.7 (5, 41)	20.8 (4, 84)	0.64	14	20.6 (9, 56)	NA
Cidofovir		22.3 (8, 31)				
**CMV treatment costs**[5]	5'061 (600, 11'025)	11'025 (2'716, 24'012)	0.14	2'891 (879, 7'969)	7'724 (839, 32'335)	0.32
Ganciclovir	4'529 (1'684, 7'374)	3'564 (710, 8'925)	0.69		3'208 (77, 8'459)	NA
Valganciclovir	1'109 (319, 2'797)	2'511 (559, 4'474)	0.02	1'068 (679, 1'598)	1'955 (559, 5'672)	0.19
Foscarnet	5'907 (1'509, 10'289)	10'306 (1'759, 32'176)	0.47	7'290	10'342 (3'770, 27'651)	NA
Cidofovir		4'243 (2'546, 6'790)	NA			
**Length of stay**[6]	47.1 (22, 109)	55.6 (8, 180)	0.47	40.3 (2, 81)	68.5 (3, 180)	0.20
From HCT to discharge[7]	34.1 (22, 62)	46.3 (8, 180)	0.25	NA	NA	NA
**Hospitalization costs**[8]	120'856 (56'496, 279'912)	142'845 (20'544, 462'240)	0.47	103'362 (5'136, 208'008)	175'822 (7'704, 462'240)	0.21
**Readmission**	5 (21.2)	12 (37.5)	0.76	3 (30.0)	5 (25.0)	1.00
>1 Readmission	2 (12.5)	4 (12.5)	1.00	0	2 (10.0)	0.54
**Letermovir**[5]	37'257 (10'543, 81'828)		NA	32'129 (1640, 64553)		NA
**All-cause 6-month mortality**	3 (18.8)	8 (25.0)	0.73	1 (10.0)	5 (25.0)	0.63
**Non-CMV viral infection**[9]	7 (43.8)	11 (34.4)	0.55	5 (50)	7 (35.0)	0.46
Herpes simplex virus 1/2	0	2 (6.3)	0.55	0	1 (5.0)	1.00
Epstein-Barr virus	3 (18.8)	1 (3.1)	0.10	3 (30.0)	4 (20.0)	0.66
Human herpes virus 6	1 (6.3)	3 (9.4)	1.00	0	1 (5.0)	1.00
Adenovirus	0	4 (12.5)	0.29	0	0	NA
BK-virus	3 (18.8)	4 (12.5)	0.67	2 (20.0)	1 (5.0)	0.25

csCMV: Clinically Significant Cytomegalovirus, D-: Donor Negative, R+: Recipient Positive, GvHD: Graft versus host Disease, HCT: Hematopoietic Cell Transplant, GvHD: Graft versus Host Disease, NA: Not Applicable.

[1] Results are presented from study inclusion and up to day 180 post-study inclusion. Numerical variables are presented as mean (range). For patients in the CMV donor negative / recipient positive group, day of study inclusion coincided with HCT day.

[2] The first documented episode of csCMV infection and or disease, for patients who had >1 episode. Seven of 9 case-patients tested for letermovir-resistance were negative. There was only one case of CMV disease in one control-patient.

[3] There were 3 case-patients who had 2 csCMV infection episodes. There were 26 and 5 control-patients with 2 and 3 csCMV infection episodes, respectively.

[4] Treatment duration represents CMV pre-emptive and targeted treatment that was administered for all documented episodes of csCMV infections/disease during the study period (from study inclusion and up to day 180 post-study inclusion). Results are presented as mean days (range).

[5] Costs are presented in US$. Results are presented as mean (range).

[6] Length of stay refers to the overall length of stay from study inclusion until day 180 post-study inclusion. For patients with >1 admissions, length of stay was calculated by adding all days of hospitalization during the study period. Results are presented as mean days (range).

[7] These results are applicable for the CMV D-R+ patient group only.

[8] Hospitalization costs during the first 6 months post study inclusion were measured according to the 2018 Swiss accounting REKOLE® system to ensure the accuracy and comparability of costs, 2018 being the most recent year of reference. The 2018 mean cost per patient per hospitalized day in an internal medicine ward was US $2,568. Estimated total hospitalization costs were calculated by multiplying the length of stay for each patient in days by US $2,568.

[9] 2 control-patients in the CMV D-R+ group had >1 non-CMV double-stranded DNA viral infection.

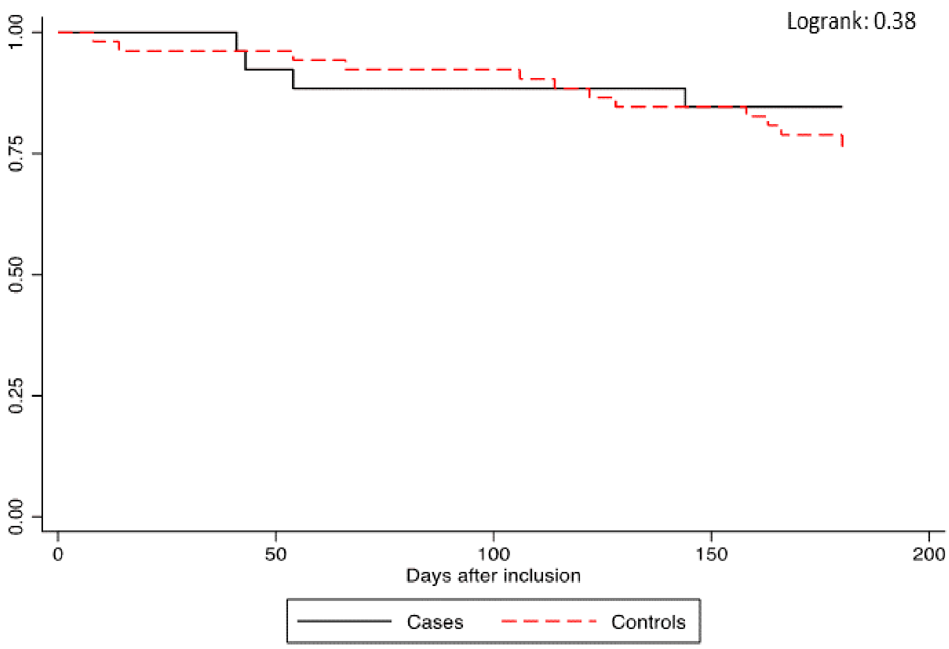

Figure S1. All-cause mortality by day 180 post study inclusion between cases and controls in the overall patient population.

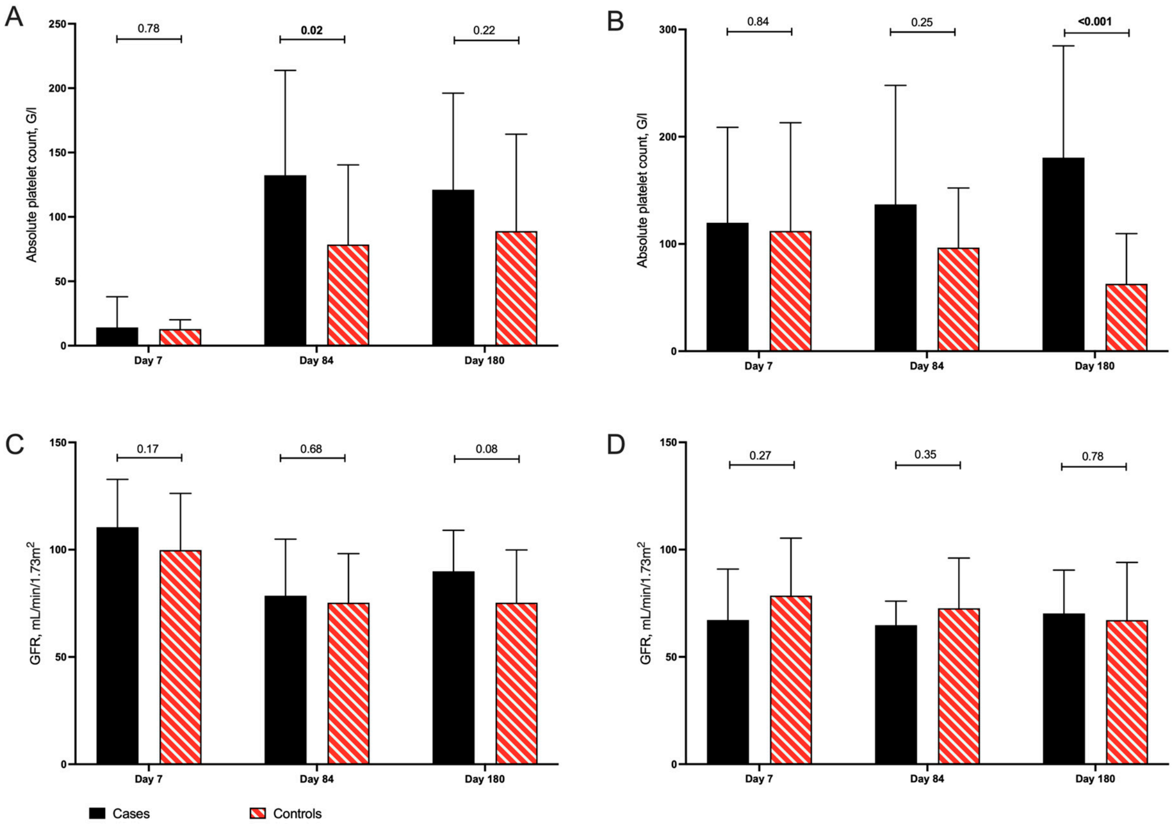

Figure S2. Distribution of platelet counts (G/l) in the D-R+ (A) and GvHD (B) groups and glomerular filtration rate (ml/min/1.73m^2) in the D-R+ (C) and GvHD (D) groups by days 7, 84 and 180 after study inclusion.
